# E-Health Interventions for Adult and Aging Population With Intellectual Disability: A Review

**DOI:** 10.3389/fpsyg.2018.02323

**Published:** 2018-11-26

**Authors:** Andrea Vázquez, Cristina Jenaro, Noelia Flores, María José Bagnato, Ma Carmen Pérez, Maribel Cruz

**Affiliations:** ^1^Facultad de Ciencias Humanas y de la Educación, Universidad Politécnica Salesiana, Guayaquil, Ecuador; ^2^Facultad de Psicología/INICO, Universidad de Salamanca, Salamanca, Spain; ^3^Facultad de Psicología, Universidad de la República, Montevideo, Uruguay; ^4^Facultad de Enfermería y Nutrición, Universidad Autónoma de San Luis Potosí, San Luis Potosí, Mexico

**Keywords:** e-health, e-therapy, intellectual disabilities, aging, systematic review

## Abstract

To answer the question about which e-health and e-therapy applications are being used with people with intellectual disabilities, we searched the PsycINFO, Medline, PubMed, ERIC, CINAHL, Scopus, Web of Science, and Cochrane databases. This is an extensive search. Inclusion criteria were academic journals and any design type that addressed the topic of interest. Studies that do not include adults or elderly, and studies that do not focus on people with disabilities but on third parties, were excluded. After an initial selection of 515 articles, 32 full-text articles were subjected to in-depth analysis leading to the final selection of 18 articles. We used the AAID framework definition of intellectual disability to analyze the dimensions explored by the selected studies and found that the majority of studies focused on the use of technology as supports to instrumental activities of daily life. The ISO classification of assistive products allowed us to identify that many e-health products are aimed at providing psychological or medical treatment. In summary, this review suggests that there is a very small number of studies focusing on the use of technology by older persons with intellectual disabilities. The studies present substantial limitations regarding generalization and replication and pay little attention to the maintenance of cognitive abilities in this population. These aspects, together with premature aging generally associated with many conditions that lead to intellectual disability, underscore the need to pay more attention to and develop e-health interventions for cognitive stimulation for this group.

## Introduction

The arrival of technology in our lives has meant changes in the way we relate to others. It has also increased our possibilities for communication, leisure and work, and it has reduced distances and physical barriers, including those related to health care (Morland et al., 2017). The development of technologies for health care is known as e-health and includes the set of technologies that are used to prevent, diagnose, and treat in healthcare environments. More specifically, according to the World Health Organization (WHO), e-Health is the application of information and communication technologies across the wide range of activities that are carried out in the health care, from diagnosis to follow-up (Vukovic et al., 2018). This definition suggests the use of information tools to support and promote the prevention, diagnosis, treatment, and monitoring of diseases and also the management of health and lifestyles, tailored to individual patients and consumers (Vukovic et al., 2018). The term ‘telehealth’ has for many years been the umbrella term in Europe used for a broad range of technologies which includes telemedicine (the sharing of medical data, including scans and visual images), e-care or m-care (which involves data transfer on a mobile basis) and telecare (Doughty et al., 2008). The terms ‘telemedicine,’ ‘telehealth,’ and ‘e-health’ are often used interchangeably by both health care providers and consumers, and It appears that the term e-health will be more popular than telemedicine or telehealth within the next ten years (Fatehi and Wootton, 2012).

Associated to e-health is m-health, that is, mobile technology used to carry out health tasks. Mobile health (m-Health) was first defined as mobile computing, medical sensors and communication technologies for healthcare (Istepanian and Al-Anzi, 2018). It has evolved since then into a major global healthcare delivery innovation and technological area. Today, smart phone m-Health applications (Apps) are increasingly connected to variety of wearable sensors and the Internet of Things (IOT) devices in variety of healthcare applications (Istepanian and Al-Anzi, 2018). In other words, e-health includes m-health, but not vice versa, necessarily.

In the field of health in adults and the elderly, e-health technologies are demonstrating their effectiveness in reducing social isolation (Khosravi et al., 2016), improving adherence to treatments (Alkhaldi et al., 2016), access to specialists (Ternullo et al., 2013; Deslich and Coustasse, 2014; Vimalananda et al., 2015), and receiving diagnosis and treatment (Norum et al., 2007). Meanwhile, m-health has been found useful for managing the health of elderly people with chronic conditions (Raphael et al., 2017), remembering medical appointments (Hasvold and Wootton, 2011), improving support for people with dementia (Jackson et al., 2016), and screening for cognitive decline (Martin-Khan et al., 2010). It is also useful for primary prevention, that is, in the promotion of healthy habits regarding food and physical activity in the general population (Goode et al., 2012). In this digital era and in the context of progressive aging, the use of e-health is also postulated as a tool to reduce costs, increase accessibility and improve the results of interventions (Kim et al., 2014; Chesser et al., 2016).

Of special interest are people with intellectual disabilities since, by definition, this group has support needs derived from their condition (Kottorp, 2008; Durbin et al., 2017), with the presence of additional needs related to potential psychological or functional impairments. Intellectual disability, according to the AAIDD (Schalock et al., 2010), is a disability that originates before the age of 18 and is characterized by significant limitations in both intellectual functioning and in adaptive behavior, which covers many everyday social and practical skills. Intellectual functioning refers to general mental capacity, such as learning, reasoning, problem solving, and so on. Adaptive behavior is the collection of conceptual, social, and practical skills that are learned and performed by people in their everyday lives. People with intellectual disabilities require different supports and, in this regard, the ISO 9999:2016 establishes a classification and terminology of assistive products for persons with disability. This classification distinguishes between assistive products for education and for training in skills, for self-care, for personal mobility and transportation, for domestic activities, for work activities and participation in employment, and for recreation and leisure, among other types of support (ISO, 2016).

Premature aging and frailty are conditions typically associated with the population with intellectual or developmental disabilities (Perkins and Small, 2006; Alcedo et al., 2017; McKenzie et al., 2017). In addition, early dementia and, hence, increased support needs, is common in people with different conditions associated to intellectual disabilities (Windley and Chapman, 2010; Furniss et al., 2012; Sutherland et al., 2014; Wark et al., 2014). In this field, e-health has proven to be useful for the empowerment of general patients, in the change of habits related to their health, and self-monitoring of their health (Clough and Casey, 2015; Wiederhold, 2015).

Although e-health appears as an opportunity, a challenge, and a right for aging people with intellectual disabilities, little is known about this topic. The Convention on the Rights of Persons with Disabilities, CRPD (United Nations, 2006) establishes in article 4 that “States should undertake or promote research and development of, and promote the availability and use of new technologies, including information and communications technologies, mobility aids, devices and assistive technologies, suitable for persons with disabilities, giving priority to technologies at an affordable cost” (section g). The current study is aligned with that statement and, to our knowledge, no literature review has been developed on this matter.

Thus, the present study provides a systematic literature review to identify the e-health technologies being used with this population, as well as their strengths and shortcomings.

## Materials and Methods

### Eligibility Criteria

For the selection of articles, the four following inclusion criteria were established; (1) The study is related to research on intellectual and/or developmental disability and technology; (2): The study includes middle age (40–64) and older population, to take into account the early aging in this population, (3) Type of design: any design (4): The article is written in English, French or Spanish. In addition, four exclusion criteria were established: (1) Studies that do not focus on people with disabilities but on third parties (e.g., caregivers, professionals, etc.). (2) Summaries and news of congresses, or editorials, or responses to other authors, etc., (3) e-health or M-health is not the focus of the paper or this technology is barely mentioned, (4) papers not focused on participants with intellectual disability, but with other primary conditions associated to cognitive impairment or cognitive decline (e.g., stroke and Alzheimer).

### Information Sources and Search Procedure

Studies were retrieved through electronic systematic literature searches, in which text words related to target population (“intellectual disabilities”) were combined through the Boolean operator “AND” with keywords and text words indicative of the target topic (“ehealth or e-health or telecare or telemedicine or telehealth”). A more specific search on “mhealth or mobile health or m-health or mobile app or mobile application” was not considered for the current study, as it exceeded the scope of the current article. The following online databases were searched: PsycINFO, Medline, ERIC, CINAHL, SCOPUS, Web of Science, PubMed, and Cochrane databases (no limit–December 2017).

### Process of Selection of the Studies

**FIGURE 1:**
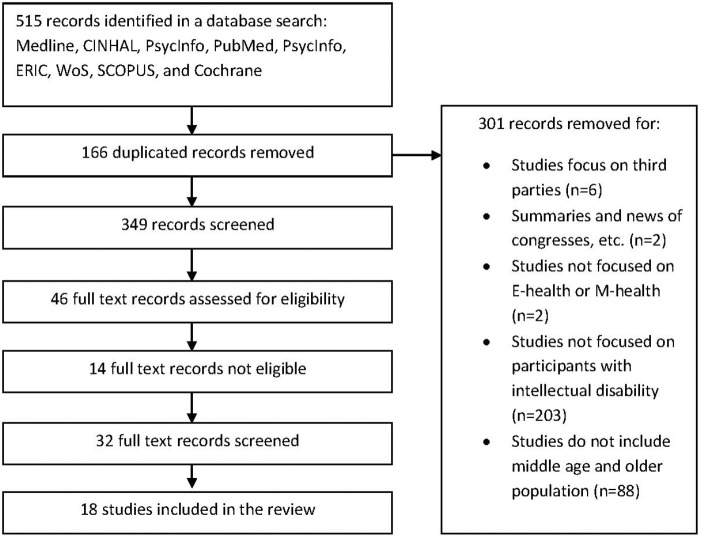
PRISMA flowchart of the selection process of the studies.

Studies were assessed on eligibility criteria by three independent reviewers (the first three authors of the current study). First, studies were examined with regard to inclusion criteria after reading the title and the abstract. When there was no agreement on inclusion between the reviewers, an additional reviewer (fourth author of the current study) was consulted to obtain agreement on included studies. This process led to the final selection of 18 articles for further analyses (see Figure 1).

### Quality Assessment and Data Extraction

Next, the methodological quality of the 18 studies was analyzed using a format adapted from Berra et al. (2008) to assess the quality of epidemiological and cross-sectional studies. Berra incorporates the recommendations of other authors like the STROBE initiative (Vandenbroucke et al., 2007). The procedure involved evaluating 27 quality indicators to determine the methodological soundness of the studies. Thus, each indicator (e.g., research question and objective, participants, groups comparison, variables definition and measurement, statistical analysis and bias risk, internal validity, results, conclusions, external validity and applicability, conflict of interests, and global quality) was rated in terms of quality (high-medium-low) and applicability (i.e., “non applicable” when adequate). Disagreements between authors were solved by discussion. Two additional reviewers (fifth and sixth authors) were involved when necessary. The analyzes indicated that 27.78% of the studies obtained a global score of high quality, while another 16.67% obtained medium quality and score, and 55.56% were scored as low quality from a methodological point of view. Nevertheless, in contrast to quantitative synthesis (i.e., meta-analysis), where the methodological quality assessment serves as criteria to identify and remove low quality studies from further data extraction and analysis, in this systematic review, the criteria was used to analyze the state of the art regarding the topic of study, instead of being an exclusion criteria. All the included empirical studies reported ethic committee approval or informed consent.

Next, a standardized form made with Microsoft Excel 2013 was used to extract data from the selected studies. In addition to data such as country, year, etc., the reviewers selected the main topic(s) of the studies (e.g., conceptual/cognitive skills, Social skills, Physical health, etc), the aims of the e-health (personalized medical treatment, training/learning skills, personal care and protection, and domestic activities), as well as the main outcomes. Table 1, in the next section, summarizes main findings that were obtained by consensus.

**Table 1:** Characteristics of the studies.

Authors	Year	Country	Participants	Domains							Aims^∗^	Topic	Outcomes
				CCS	SS	BS	IS	PH	MH	E			
Beyer and Perry	2013	United Kingdom	Literature review					×			2	Assistive technology	Independence improvement
de Wit et al.	2015	Netherlands	*n* = 30 adults with ID or MI		×		×				4	Web-based support for daily functioning	Independence improvement
Deverson et al.	2015	Netherlands	*n* = 2 adults with ID and *n* = 2 relatives		×		×				2, 5	Videophone to improve communication	Usability and Independence improvement
Gentile et al.	2017	United States	Literature review						×		1	Telepsychiatry	Cost reduction and improved mental health
Hall et al.	2011	United Kingdom	*n* = 20 adults with ID				×				2, 5	Virtual Realty to Provide Health Care Information	Accessibility, usability and control of mental health
Haymes et al.	2015	United States	Literature review	×			×	×			1, 2	Technology for managing healthcare needs	Usability and Improvement of physical health
Jonker et al.	2015	Netherlands	*n* = 1 adult with ID						×		1	Technology assisted therapy	Usability and improvement of mental health
Krysta et al.	2017	Poland	Literature review				×		×		1	Telepsychiatry	Usability and improvement of mental health
Neumeier et al.	2017	United States	*n* = 70 adults with ID (*n* = 35 control group, and *n* = 35 experimental group)			×	×	×		×	1, 2	Weight control	Usability and improvement of physical health
O’Hara et al.	2008	United States	*n* = 36 adults with ID			×		×			1, 2	Oral health with technologies	Usability and improvement of physical health
Perry and Beyer	2012	United Kingdom	*n* = 5 adults with DE, and *n* = 5 adults with ID (plus carers)				×	×	×		1, 2	Assistive technology and telecare	Usability and health control
Perry et al.	2012	United Kingdom	*n* = 91 adults with ID (*n* = 63 experimental group)			×	×	×			1, 2, 3, 4, 5	Tele care for independent living	Independence improvement and health control
Sheehan and Hassiotis	2017	United Kingdom	Literature review	×	×	×	×	×	×	×	1	Telepsychiatry	Accessibility, usability and control of mental health
Szeftel et al.	2012	United States	Literature review	×			×		×		1	Telepsychiatry	Improvement of mental health
Taber-Doughty et al.	2010	United States	*n* = 4 adults with ID	×		×	×	×			1, 2, 3, 4, 5	Tele care for independent living	Usability and Independence improvement
Temple et al.	2010	Canada	*n* = 19 adults with ID	×							1	IQ diagnosis by technology	Diagnostic accuracy of capabilities
van Dooren et al.	2013	Australia	*n* = 4 adults with ID, *n* = 4 family members, *n* = 2 supporters					×		×	1	e-health records	Accessibility, usability and control of physical health
Wilkie	2010	United Kingdom	*n* = 2 adults with ID, *n* = 2 youth with ID								1	Assistive technology and telecare	Independence improvement

*ID, intellectual disabilities; MI, mental illness; DE, dementia; CCS, conceptual/cognitive skills; SS, social skills; BS, basic daily living skills; IS, instrumental daily living skills; PH, physical health; MH, mental health; E, environment (barriers/participation); ^∗^1, measuring, supporting, training, or replacing body functions (ISO 04); 2, education and training in skills (ISO 05); 3, self-care care activities and participation (ISO 09); 4, domestic activities and participation in domestic live (ISO 15).*

## Results

As summarized in Table 1, the United States and the United Kingdom, followed by the Netherlands, lead the studies on this matter. This topic is a subject of interest since a decade ago, although most of the studies are more recent. Six of the 18 studies are theoretical in nature (i.e., qualitative synthesis or literature reviews). Twelve studies were empirical in nature, and most of them have a small sample size (Mean = 27, range: 1–91).

Using the intellectual disability conceptual framework proposed by the AAIDD, we have classified the studies according to the skills they deal with: conceptual, social, or practical. Also, physical or mental conditions as well as environmental conditions that may act as barriers or facilitators for participation have been identified. The analyzes revealed (see Table 1) that the majority of the studies focused on technology for carrying out instrumental activities of daily life (IS: 61.11%), as well as additional needs related to physical health (PH: 50%). Mental health and its relationship with technologies was identified in a lower percentage of studies (MH: 33.3%). A smaller proportion of studies focus on the use of technologies in relation to basic daily life skills (BS: 27.8%), and the intervention in conceptual/cognitive skills (CCS: 27.8%). Social skills and their relationship with technologies are addressed in a smaller number of studies (SS: 16.67%) as were studies focused on the environment and its accessibility (E: 16.67%).

Next, using the ISO assistive products classification framework, we classified the studies according to theirs aims. The analyzes showed that the vast majority of technologies are aimed at providing psychological or medical treatment (77.5%), followed by those aimed at education and training in skills (50%), and aid for communication and information management (22.2%). A small number of studies focus on technology for carrying out domestic activities (16.7%) and for self-care (11.1%). Other possible functions, such as a support product for the improvement of employment and training, recreation or improvement of the environment, are not considered in the studies. Four out of the 18 articles deal with reviews on telepsychiatry services for people with intellectual disabilities (Szeftel et al., 2012; Gentile et al., 2017; Krysta et al., 2017; Sheehan and Hassiotis, 2017), and one article shows the utility of technology assisted therapy for anxiety treatment (Jonker et al., 2015). The analysis of general (Beyer and Perry, 2013) and ethical issues associated to technology use with people with intellectual disabilities is another topic in the selected studies (Perry et al., 2009; Perry and Beyer, 2012). Technology for managing healthcare needs of people with intellectual disabilities, either in general (Wilkie, 2010; Hall et al., 2011; van Dooren et al., 2013; Haymes et al., 2015) or associated to specific health needs, such as weight control (Neumeier et al., 2017), or oral health (O’Hara et al., 2008) are also included. In addition, two articles deal with telecare as a support tool for promoting independent living (Taber-Doughty et al., 2010; de Wit et al., 2015), and one article shows the utility of videophones to improve communication (Deverson et al., 2012). Finally, one study analyzes the applicability of technology for diagnosis purposes on intellectual disabilities (Temple et al., 2010).

## Discussion

The current review has identified that the majority of the studies focus on the applicability of e-health interventions to improve the performance in daily life activities. More specifically, Web-based support for daily functioning and independent living, by improving communication, providing health care Information and meeting both physical (e.g., weight control), and mental (i.e., telepsychiatry) healthcare needs. Although these uses match the definition of e-health, what is noteworthy the scarcity of studies focused on the use of e-health technology by people with intellectual disabilities, with even fewer for the elderly population with intellectual disabilities. In addition, a large number of studies are qualitative, narrative review with methodological limitations in terms of the possibility for generalization, replication, etc. Empirical studies usually include very small and incidental samples, indicating the existence of a clear gap requiring for further investigation.

A large number of the selected papers (O’Hara et al., 2008; Taber-Doughty et al., 2010; Hall et al., 2011; Deverson et al., 2012; Perry and Beyer, 2012; van Dooren et al., 2013; Haymes et al., 2015; Jonker et al., 2015; Krysta et al., 2017; Neumeier et al., 2017; Sheehan and Hassiotis, 2017) mention problems related to the accessibility and usability of these technologies in the population of interest. One of the issues is the scarcity of studies with this population (Krysta et al., 2017; Neumeier et al., 2017) so, as Sheehan and Hassiotis (2017) state, the application of new technologies for people with intellectual disability seems to have been largely neglected. In addition, other studies stress the need for extra support in order to guarantee a successful use (e.g., Hall et al., 2011; Deverson et al., 2012), and the challenges poses by different levels of support needs and above all for those with the most severe disabilities, which calls for social validation strategies (Taber-Doughty et al., 2010; Hall et al., 2011; Deverson et al., 2012; van Dooren et al., 2013; Haymes et al., 2015; Jonker et al., 2015; Neumeier et al., 2017). Likewise, technical issues that may reduce the usability of the proposed technologies are mentioned in several studies (O’Hara et al., 2008; Taber-Doughty et al., 2010; Deverson et al., 2012), so using principles of universal design to improve access (Sheehan and Hassiotis, 2017) is advisable. These findings underscore the need to guarantee equity in access to these technologies for health care, maintaining the principles emanating from the CRPD (United Nations, 2006).

Most of the studies focus on the use of these technologies for the control of associated psychiatric conditions, as well as for the maintenance and control of physical health. In contrast, little attention is paid to the maintenance of cognitive abilities in this population. Instead, existing studies focuses on cognitive disturbances or disorders and its diagnosis (Temple et al., 2010) and treatment (Szeftel et al., 2012; Haymes et al., 2015; Sheehan and Hassiotis, 2017), as well as on the supports associated to cognitive limitations (Taber-Doughty et al., 2010). In contrast to the numerous studies that have evaluated the effectiveness of cognitive training programs in older adults, there is a scarcity of studies with aging adults with intellectual disabilities (Perkins and Small, 2006). More studies focused on normative and pathological aging in this group, as well as the use of adapted e-health for diagnosis and treatment purposes are very advisable. Alternatives such as serious games, virtual reality and social networks are promising areas due to the innumerable benefits they can bring to this group in the field of health (Chang et al., 2013; Saridaki and Mourlas, 2013; Borrelli and Ritterband, 2015; Yan et al., 2015).

## Author Contributions

All authors had contributed to the conception and design of the study, data collection, and/or analysis and interpretation of the data; had drafted the paper or revised it critically; had approved the final version submitted for publication; had complied with APA ethical standards throughout the whole process; and had made substantial contributions to the field of e-health and intellectual disabilities.

## Conflict of Interest Statement

The authors declare that the research was conducted in the absence of any commercial or financial relationships that could be construed as a potential conflict of interest.
